# Severe Asthmatic Responses: The Impact of TSLP

**DOI:** 10.3390/ijms24087581

**Published:** 2023-04-20

**Authors:** Efthymia Theofani, Aikaterini Tsitsopoulou, Ioannis Morianos, Maria Semitekolou

**Affiliations:** 1 Cellular Immunology Laboratory, Center for Basic Research, Biomedical Research Foundation of the Academy of Athens, 11527 Athens, Greece; 2 Host Defense and Fungal Pathogenesis Lab, School of Medicine, University of Crete, 71110 Heraklion, Greece; 3 Institute of Molecular Biology and Biotechnology, Foundation for Research and Technology, 71300 Heraklion, Greece; 4 Laboratory of Immune Regulation and Tolerance, School of Medicine, University of Crete, 71110 Heraklion, Greece

**Keywords:** severe asthma, asthma endotypes, biologics, thymic stromal lymphopoietin (TSLP)

## Abstract

Asthma is a chronic inflammatory disease that affects the lower respiratory system and includes several categories of patients with varying features or phenotypes. Patients with severe asthma (SA) represent a group of asthmatics that are poorly responsive to medium-to-high doses of inhaled corticosteroids and additional controllers, thus leading in some cases to life-threatening disease exacerbations. To elaborate on SA heterogeneity, the concept of asthma endotypes has been developed, with the latter being characterized as T2-high or low, depending on the type of inflammation implicated in disease pathogenesis. As SA patients exhibit curtailed responses to standard-of-care treatment, biologic therapies are prescribed as adjunctive treatments. To date, several biologics that target specific downstream effector molecules involved in disease pathophysiology have displayed superior efficacy only in patients with T2-high, eosinophilic inflammation, suggesting that upstream mediators of the inflammatory cascade could constitute an attractive therapeutic approach for difficult-to-treat asthma. One such appealing therapeutic target is thymic stromal lymphopoietin (TSLP), an epithelial-derived cytokine with critical functions in allergic diseases, including asthma. Numerous studies in both humans and mice have provided major insights pertinent to the role of TSLP in the initiation and propagation of asthmatic responses. Undoubtedly, the magnitude of TSLP in asthma pathogenesis is highlighted by the fact that the FDA recently approved tezepelumab (Tezspire), a human monoclonal antibody that targets TSLP, for SA treatment. Nevertheless, further research focusing on the biology and mode of function of TSLP in SA will considerably advance disease management.

## 1. Severe Asthma

### 1.1. Background

Asthma represents the most common chronic lung disease that affects up to 18% of the population of all age groups in different countries. Notably, during the last decades, its prevalence has increased, especially among children [1]. Asthma is characterized by recurring symptoms of reversible airflow obstruction, airway hyperresponsiveness (AHR) to otherwise harmless environmental antigens, and airway inflammation. Several parameters such as allergen or irritant exposure, viral respiratory infections, and even exercise, climate changes, or stress are responsible for the variations and intensity of these symptoms [1]. The resolution of clinical symptoms and airflow limitation may occur spontaneously or after medication in some patients and can last for weeks or even months [2]. Asthmatic individuals are characterized by extensive heterogeneity in their clinical features and phenotypes [2]. A percentage of approximately 5–10%, of the total asthma population exhibit severe disease exacerbations despite being treated with high doses of either inhaled or systemic corticosteroids, often in combination with a second line of medication [2,3]. These patients suffer from uncontrolled severe asthma (SA), which, for some of them, could be life-threatening [4,5]. Severe asthmatics comprise a small percentage of total asthma patients. Nevertheless, 50% of total healthcare costs are attributed to their treatment, representing a major healthcare and economic burden worldwide [6,7]. In terms of lung biology, SA is described by structural changes of the airway wall, particularly pronounced thickening, leading to excessive narrowing of the airways and fixed airflow obstruction [6,7]. To elaborate on SA divergency, the concept of asthma endotyping developed [8,9,10]. Two asthma endotypes have been proposed, based on the type of the immune cell responses that are responsible for disease pathogenesis. Type 2 asthma is associated with T helper 2 (Th2) cell-mediated inflammation, while non-type 2 asthma is predominantly characterized by Th1 and/or Th17-cell mediated inflammation [11,12,13].

### 1.2. Severe Asthma Endotypes

#### 1.2.1. Type 2 Asthma

Several studies have shown that certain allergens with proteolytic activity are able to invade lung epithelium by disrupting the tight junctions interconnecting epithelial cells and stimulate dendritic cells (DCs) via interacting with protease activated receptors expressed on their surface [14]. Moreover, several allergens and airborne particulates that contain microbial components interact with Toll-like receptors (TLRs), NOD-like receptors (NLRs), and other pattern recognition receptors expressed on DCs and airway epithelial cells, and serve as “danger signals” initiating T cell responses [14]. DCs exposed to allergens interact with naive CD4^+^ T cells, initiating their activation and differentiation towards Th1, Th2, Th9 or Th17 cells, based on the type and dose of allergen and the cytokine repertoire in the microenvironment [14]. In the presence of type 2 cytokines, such as IL-4, IL-5, IL-9 and IL-13, naive CD4^+^ T cells differentiate into Th2 effector cells and migrate to the asthmatic airways where they secrete cytokines and propagate cardinal features of asthma. With the latter, we mostly refer to mucus production, subepithelial fibrosis, airway remodeling and AHR [15]. The release of Th2 cytokines in the inflamed airways results in the recruitment of mast cells, basophils and eosinophils as well as other effector cells in addition to isotype switching of B cells, which start producing allergen-specific IgE [15]. Additionally, Th9 cells intensify allergic airway inflammation (AAI) through the productions of copious amounts of IL-9, which mediates mast cell activation [16,17]. In more detail, it has been demonstrated that IL-9 produced by Th9 cells and type 2 innate lymphoid cells (ILC2s) leads to the secretion of IL-2 by mast cells, further expanding ILC2s, which in turn propagate Th9 cell activation [18]. Of clinical relevance, heightened numbers of Th9 cells were observed in the periphery of subjects allergic to House Dust Mite (HDM) or pollen and correlated with their respective IgE levels [19]. Furthermore, the percentage of IL-9-producing T lymphocytes was increased in the bronchoalveolar lavage (BAL) of asthmatic individuals [20]. An important group of cytokines termed “alarmins,” which includes IL-25, IL-33, and thymic stromal lymphopoietin (TSLP) along with a broad spectrum of chemokines (e.g., eotaxins, RANTES, TARC), are secreted by the asthmatic airway epithelium upon exposure to allergens, pollutants, viral, fungal, and bacterial components and trigger Th2 cell polarization [14]. Additionally, ILC2s are activated in response to signaling from the “alarmins” [21] and produce IL-5, IL-13, and prostaglandin (PGD2) [22], further propagating Th2-cell-mediated responses in the airways and linked disease.

#### 1.2.2. Biomarkers for Type 2 Asthma

**Figure 1:**
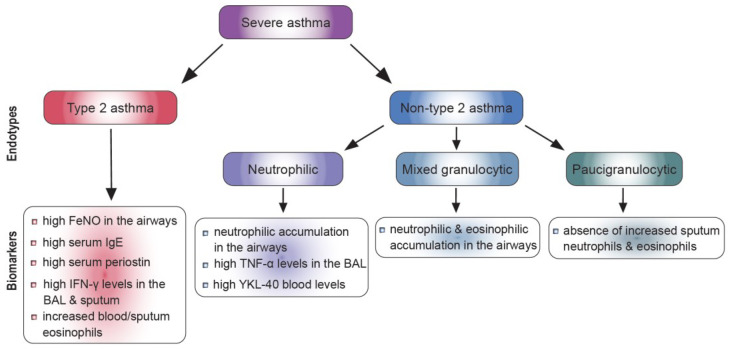
SA endotypes and biomarkers.

Several biomarkers of type 2 inflammation, such as Fractional exhaled Nitric Oxide (FeNO), serum IgE, blood or sputum eosinophils, and serum periostin, have been used to discriminate between type 2-high and low asthma phenotypes and predict the response to therapies targeting type 2 cytokines [23] (Figure 1). Eosinophils play a vital role in sustaining and enhancing chronic inflammatory asthmatic responses [12]. In particular, enhanced eosinophilic numbers have been detected in the periphery of asthmatic patients demonstrating severe disease exacerbations and poor overall asthma control [12]. FeNO is closely related to IL-13-mediated and corticosteroid-responsive bronchial inflammation since IL-13 is shown to activate inducible nitric oxide synthase, and as a result, increase the production of FeNO in the airways [12]. The majority of asthmatics display an allergic phenotype, which is characterized by allergen-specific IgE production and heightened total IgE levels. Notably, allergen-specific IgE antibodies are pivotal for the initiation and propagation of the inflammatory processes that take place in the airways in atopic asthma [12]. Finally, periostin, an extracellular matrix protein mainly secreted by bronchial epithelial cells in response to IL-4 and IL-13, denotes another key biomarker for severe eosinophilic type 2 asthma [24]. Periostin has been implicated in airway remodeling, sub-epithelial fibrosis, eosinophil infiltration, and mucus secretion, and its serum concentration denotes one of the major indicators of eosinophilic airway inflammation [25]. Clustering studies have demonstrated that except for Th2 inflammation, other inflammatory mediators implicated in SA pathogenesis are responsible for the failure of SA patients to respond to corticosteroid (CS) treatment [8,26]. In fact, increased percentages of CD4^+^ IFN-γ^+^ T cells, along with higher levels of IFN-γ, at the mRNA and protein level, were detected in the BAL of SA patients in comparison to mild-moderate asthmatics (MMA) [27]. In accordance with the above-mentioned studies, elevated IFN-γ mRNA levels were also found in the sputum and lung tissue specimens of SA patients [28,29]. The major triggers of IFN-γ-mediated responses associated with enhanced disease severity and asthma exacerbations are persistent viral (mostly rhinoviruses) and bacterial infections (*C. pneumoniae*, *S. pneumoniae*, *M. pneumoniae*, *H. influenzae*, *M. catarrhalis*, and *S. aureus*) [30].

#### 1.2.3. Non-Type 2 Asthma

According to the type of immune cells that infiltrate the airways, non-type 2 asthma can be divided into neutrophilic, mixed granulocytic and paucigranulocytic (PGA) endotypes [31]. However, the pathophysiology of non-type 2 asthma remains less well-defined compared to that of type 2 asthma. Non-type 2 asthma is mainly characterized by the lack of type 2 biomarkers and the prevalence of neutrophils and Th17 cells in the airways [32]. Recent studies have revealed that immature blood neutrophils, as well as activated mature ones, are linked to asthma pathogenesis [33,34]. Additionally, other studies have demonstrated that neutrophils obtained from the sputum of asthmatic patients had a greater anti-apoptotic activity compared to healthy controls, and that this characteristic increased when asthma severity was higher [35]. Moreover, Th1 inflammatory cytokines are associated with neutrophilic asthma. In one report, BALF from severe asthmatic patients showed higher Th1 cell and neutrophil numbers accompanied by elevated IFN-γ levels, a signature Th1 cytokine [27]. Additionally, IL-17 is implicated in the development of neutrophilic airway inflammation in asthma. IL-17 levels in bronchial biopsies are associated with airway neutrophil infiltration and are enhanced in patients with severe asthma compared with those with milder disease [36]. Notably, polyclonally stimulated CD4^+^ T cells isolated from the airways of SA patients produced considerable amounts of IL-17 and IL-22 [37,38]. Another study illustrated that human bronchial epithelial cells (HBECs) and venous endothelial cells secreted more IL-8 upon in vitro culture in the presence of IL-17 [39]. In addition, conditioned medium from IL-17-treated HBECs promoted the migratory capacity of human neutrophil in vitro [39]. Concordantly, heightened IL-17 gene expression has been detected in cells obtained from the sputum of SA patients compared to healthy controls [40]. Additionally, a positive correlation between disease severity and IL-17 levels in the periphery of SA patients has been reported [41]. Recently, an appealing study demonstrated that an IL-4Rα polymorphism found in SA patients was associated with the skewing of regulatory T cells (Tregs) to Th17-like cells, characterized by increased secretion of IL-17 [42]. Nevertheless, targeting IL-17 axis did not provide the anticipated therapeutic outcome in SA patients, as opposed to anti-type 2 cytokine therapy, implying that targeting pathogenic Th17 cells would be more appropriate [43,44].

In regards to PGA asthma, this endotype is not accompanied by enhanced eosinophil or neutrophil detection in sputum, but instead is characterized by low-grade bronchial inflammation linked to the dysfunction airway smooth muscle (ASM) cells, tenacious airflow obstruction and AHR [45,46]. Furthermore, in PGA asthma, contrary to the other two endotypes, factors involved in oxidative stress, such as matrix metalloproteinases, neutrophil elastase and galectin-3, cannot be considered as biomarkers, as their expression remains unaltered [47,48,49,50]. Additionally, reduced levels of FeNO were detected in PGA patients compared to those with eosinophilic asthma [51]. Notably, a recent study showed that PGA represents the most prevalent endotype in pediatric asthma [52]. Moreover, PGA asthmatics display resistance to corticosteroid treatment, regardless of the dose administered [53]. Taking into account that the symptoms that prevail in PGA are mainly attributed to alterations in the ASM phenotype and/or neuronal dysregulation, therapeutic regimes targeting ASM responses might be beneficial for these patients [54]. Specifically, the application of bronchial thermoplasty is believed to reduce the mass of ASM, even though the mode of function of this approach remains ill-defined [54]. Furthermore, mediators of subepithelial basement membrane thickening and ASM malfunction could be envisioned as biomarkers and guide the design of novel therapeutic regimes for PGA [47].

#### 1.2.4. Biomarkers for Non-Type 2 Asthma

To date, biomarkers of type 2-low or neutrophilic asthma have not been defined. So far, heightened numbers of eosinophils have been reported to be present concomitantly with neutrophilic accumulation in the airways of SA patients [55] (Figure 1). Moreover, although measuring eosinophil numbers can predict eosinophilic asthma, the amount of blood neutrophils does not recapitulate the proportion of neutrophils in the sputum [56,57]. Recently, the chitinase-like protein YKL-40 was proposed to be used as a biomarker for non-type 2 neutrophilic asthma [58]. Nevertheless, relating the measurement of YKL-40 with several other clinical parameters may deliver a more valid strategy for classifying non-type 2 asthma. Additionally, tumor necrosis factor (TNF-α) has been shown to have an essential role in non-type 2 asthma by acting directly on smooth muscle cells of the respiratory tract or by modifying the release of the cysteinyl leukotrienes LTC4 and LTD4 [59]. Importantly, it has been reported that TNF-α levels in the BAL and TNF-α mRNA expression and protein levels in bronchial biopsy specimens were increased in SA compared to MMA patients [60]. Notably, inhalation of recombinant TNF-α from healthy individuals resulted in the development of AHR and bronchial neutrophilia [61,62]. Several clinical trials using anti-TNF-α therapy have allowed the investigation of the role of this cytokine in vivo [63]. Early studies revealed an improvement in quality of life, lung function and AHR and a reduction in exacerbation frequency in asthmatic patients treated with anti-TNF-α therapy [63]. Nevertheless, it should be taken into consideration that there is significant heterogeneity in patients’ responses, suggesting that the benefit from anti-TNF-α therapy is likely to be applicable to a small subgroup of SA patients.

### 1.3. Targeted Therapies for Severe Asthma

SA disease management has been considerably improved with the development of innovative therapeutic approaches that would not have been possible without the thorough investigation of the cellular and molecular mechanisms underlying SA pathophysiology. In fact, antibodies that target mediators implicated in SA pathophysiology are already being employed as a first-line treatment. In this direction, therapeutic regimes for patients with uncontrolled allergic asthma have been reinforced with omalizumab, a monoclonal antibody which aims at human IgE [64]. Moreover, monoclonal antibodies against IL-5 (reslizumab, mepolizumab), IL-5R (benralizumab), and IL-4R (dupilumab) have become add-on treatments for uncontrolled type 2 eosinophilic asthma [64]. Nevertheless, these therapies cannot efficiently manage disease symptoms in individuals with non-type 2 asthma, as well as a significant proportion of individuals with severe allergic and/or eosinophilic asthma [64]. Of note, these monoclonal antibody therapies are not devoid of adverse effects (AEs). In one such case, omalizumab has been related to anaphylaxis at a rate of 0.09%, which most of the times occurs within 2 h after the first dose and 30 min after subsequent doses, highlighting the need for patient monitoring [65,66]. Furthermore, omalizumab has also been attributed to a higher incidence of cardiovascular and cerebrovascular AEs [67]. Mepolizumab, an anti-IL-5 monoclonal antibody approved for eosinophilic asthma, has been related to headaches, back pain, injection site reactions and fatigue [68]. In the case of reslizumab, another FDA-approved antibody targeting IL-5, its most noteworthy AEs are anaphylaxis at a rate of 0.3%, musculoskeletal and oropharyngeal pain and enhanced serum creatinine kinase [69]. Regarding benralizumab, a recently FDA-approved anti-IL-5R antibody, there have been no documented AEs apart from nasopharyngitis and injection site reactions [70]. In asthmatic individuals receiving dupilumab, a monoclonal antibody against the common receptor subunit for IL-4 and IL-13, AEs include nasopharyngitis, headaches, and injection site reactions [71]. Notably, there is a number of candidates, such as, IL-25, IL-6, TNF-like ligand 1A, CD6 and activated cell adhesion molecules, which are currently being explored as therapeutic targets and which might occur in future clinical trials [64]. The outcome of such clinical trials will be of great value as they may lay the ground for novel treatment types that will effectively replace the existing ones and result in efficient management of SA. Another important cytokine, IL-33, induces airway hyperresponsiveness through IL-13 release from mast cells and ILC2 [72,73]. A phase II trial has shown that the anti-IL-33 monoclonal antibody (REGN3500) was able to improve the control of severe asthma, but its therapeutic effects did not prove to be better than those induced by dupilumab [74]. Additionally, an anti-interleukin-23p19 monoclonal antibody, Risankizumab, was not beneficial in SA, resulting in a higher annualized rate of asthma worsening in patients compared to placebo treatment [75]. Targeting IL-1β with IL-1β antibodies or recombinant IL-1βR antagonist, such as canakinumab and anakinra respectively, is under clinical investigations, although with low efficacy [76,77]. One antibody against TSLP, which prevents TSLP binding to its receptor complex, is under clinical trials with positive results in severe asthmatics so far [78]. In this first part of the review, we provided an in-depth characterization of SA pathophysiology and a significant piece of knowledge regarding the currently available endotype-based disease biomarker. In the next part of this review, we will discuss in more detail the established and under evaluation therapeutic approaches for SA management, focusing on the essential role of TSLP in asthma regulation. Moreover, we will go over elegant studies in human and experimental SA, pertinent to the role of TSLP in the initiation and propagation of asthmatic responses. Finally, we will discuss the importance of further research centered on the biology and mode of function of TSLP in SA.

## 2. TSLP and Asthma

### 2.1. TSLP Signaling

In humans, two variants of TSLP are detected [79]. The long form (lfTSLP) and the short form (sfTSLP) of TSLP share the same carboxy-terminus, but in the case of sfTSLP, transcription is initiated from a promoter residing in intron 2, resulting in a 63 amino acid-length protein instead of 159 that is the size of lfTSLP [79]. The sfTSLP is constitutively expressed by the airway epithelium, lung fibroblasts and keratinocytes and its expression remains unaltered during inflammatory responses, whereas the lfTSLP is activated upon TLR and TNF-α stimulation [79,80,81,82,83]. In terms of their functional properties, antibacterial and anti-inflammatory functions are attributed to sfTSLP, whereas pro-inflammatory functions are attributed to lfTSLP [83,84,85,86,87]. TSLP signals through a heterodimeric receptor composed of TSLPR (a type I cytokine receptor encoded by Crlf2) and the IL-7 receptor α-chain (IL-7Rα) [88,89,90]. This receptor is expressed by several immune and non-immune cell types, such as DCs, macrophages, mast cells, basophils, T cells, epithelial cells and neurons [91]. The JAK1 and JAK2 kinases are activated via the IL-7Ra and TSLPR receptor subunits, respectively. JAK1 and JAK2 induce signal transducer and activator of transcription 5A (STAT5A) and STAT5B, ultimately leading to the production of pro-inflammatory cytokines IL-4, IL-5, IL-9 and IL-13 [92,93]. Although signaling through the combination of TSLPR and IL-7Rα applies for lfTSLP, given the truncated nature of sfTSLP, it merits further investigation whether the latter uses the same or an alternative signaling pathway.

### 2.2. Cellular Sources and Responders of TSLP

**Figure 2:**
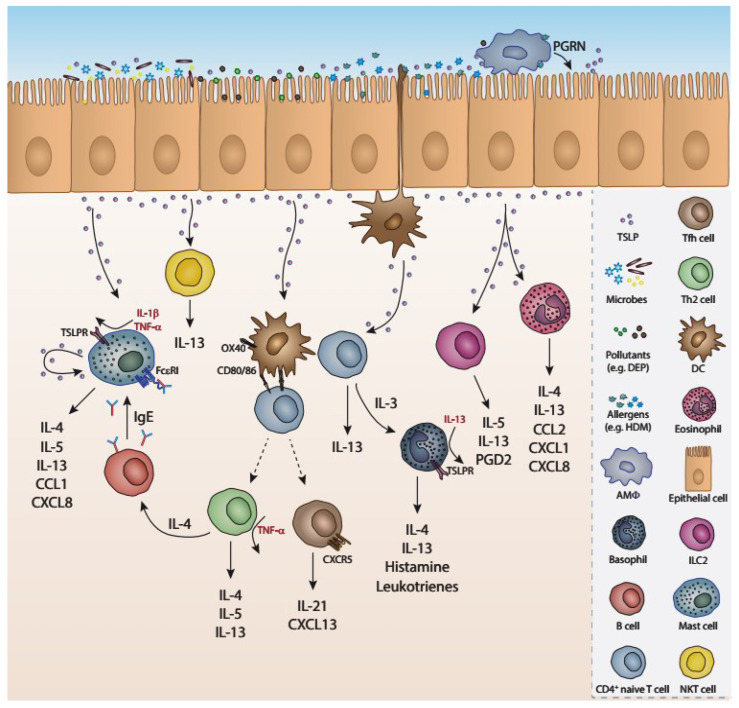
Cellular sources and targets of TSLP. Several stimuli including microbes, pollutants, allergens, proteases and cytokines such as IL-4, IL-5, IL-13, and the macrophage-derived progranulin trigger the production of TSLP from the epithelium. TSLP acts on a variety of cells, including DCs, ILCs CD4 T cells mast cells basophils, etc., and upregulates the expression of co-stimulatory molecules (CD80, CD86, OX40). It also induces the secretion of a plethora of cytokines (IL-4, IL-5, IL-13) and chemokines (CCL1, CXCL8, CXCL1, CXCL13), further amplifying the asthmatic responses.

TSLP was initially detected in the supernatant of a thymic cell line. The primary roles attributed to TSLP were the long-term maintenance of B cell line growth and the support of the proliferation of unfractionated thymocytes after polyclonal stimulation [94,95]. A wide range of cell types of the immune system express or respond to TSLP, underlying the important role of this cytokine in a plethora of biological processes [86,96] (Figure 2). Over the past few years, an important number of studies have revealed that TSLP is an essential factor of type 2 inflammation, both in humans and rodents [91]. TSLP is a pleiotropic cytokine expressed by epithelial and stromal cells in the lung, skin and gastrointestinal tube (or alimentary tract) serving thus as part of the tissue homeostatic and inflammatory mechanisms [94,96]. TSLP, along with the epithelium-derived cytokines IL-25 and IL-33, exerts crucial roles in the development of allergic diseases such as atopic dermatitis, food-hypersensitivity and allergic asthma. These alarmins act as “danger” sensors in the airways in conditions of direct damage to the epithelium or during allergic disorders, including severe asthma [97,98]. Many other stimuli can trigger epithelial cells to secrete TSLP, such as TLR2 and TLR3, NLR, helminth infection, pro-inflammatory cytokines, proteases, such as trypsin and papain [94,99,100,101,102], but also viruses, such as respiratory syncytial virus (RSV), rhinovirus [103,104,105], influenza virus and lymphocytic choriomeningitis virus [106]. Some of the positive regulators of TSLP production include the cytokines IL-4 and IL-13, TNF-α in combination with IL-1β and IL-25, as well as progranulin (PGRN), derived from murine macrophages in the airways [107,108]. In addition, the cross-linking of IgE to its FcεRI receptor in mast cells results in TSLP secretion [99]. On the contrary, IFN-γ and IL-17 act as inhibitors of TSLP expression [109], along with β2-adrenoceptor agonists and glucocorticoids [110].

Although initially identified to enhance the growth and proliferation of B cells and thymocytes [111,112], TSLP was later considered as an ‘alarmin’ with pleiotropic functions in a plethora of cell subsets, including DCs, ILCs, CD4^+^ T cells, neutrophils, mast cells, basophils and eosinophils [113,114,115,116]. More specifically, in humans, TSLP acts on DCs during inflammation and induces them to express the co-stimulatory molecules OX40 ligand (OX40L), CD80 and CD86, which participate in the proliferation, expansion and differentiation of CD4^+^ T cells into Th2 pro-inflammatory cells. The latter produce IL-4, IL-5, IL-13 and TNF-α soumelis [99,116]. Additionally, TSLP-activated DCs act directly on naïve CD4^+^ T cells via the co-stimulatory molecule OX40L and trigger them to differentiate into T follicular helper cells (Tfh), expressing CXCR5, IL-21, CXCL13 and BCL6. The latter subsequently stimulate memory B cells to secrete IgG and IgE, both of which have been associated with allergic diseases in humans [117]. Murine studies have shown that TSLP-primed DCs also stimulate CD4^+^ T cells to express IL-3, which then recruits basophils to produce IL-4. This OX40L-IL-3 axis is essential in driving the Th2 inflammation, characterized as a ‘DC-T-Baso-T’ cellular cascade [118].

Basophils produce the type 2 cytokines IL-4, IL-13 and pro-inflammatory factors such as histamine and leukotrienes [119]. Upon maturation, basophils express TSLPR, especially in the presence of IL-13 [120]. Asthmatic patients exhibit an increase in TSLPR expression on basophils in the airways after allergen challenge [121,122]. Furthermore, in patients with allergic asthma, stimulation of peripheral basophils with TSLP results in upregulation of the activation marker CD203c, type 2 cytokine production, histamine release and eotaxin-mediated cellular migration responses [123]. TSLP has also been found to act directly on naïve CD4^+^ T cells and differentiate them into IL-13^+^ cells, indicating an essential role for this cytokine in T cell activation and propagation of type 2 inflammation [91,124,125]. Furthermore, TSLP induces eosinophils, NKT cells, mast cells, macrophages and airway smooth muscle cells to express Th2 cytokines and chemokines during inflammatory diseases [94,126,127,128]. Human eosinophils also express TSLPR and IL-17Ra, and this expression is boosted by TNF-α and IL-13 [129]. Moreover, TSLP is implicated in several functions of eosinophils such as upregulation of adhesion molecules, migration to sites of inflammation, and cytokine and chemokine secretion [99]. TSLP also triggers airway smooth muscle cells to release the pro-inflammatory cytokine IL-6 and the CC/CXC chemokine IL-8 (CXCL8 and eotaxin-1/CCL11) [130]. In an experimental asthma model, it was revealed that NKT cells express TSLPR and IL-7 receptor, and TSLP was shown to directly act on NKT cells and induce the production of IL-13 and thus to increase airway hyperactivity [131].

TSLP acts directly on group 2 ILCs, both in humans and mice, to produce high levels of IL-5 and IL-13 independent of antigen-presenting cells and thus promotes type 2 cytokine–associated skin inflammation [132,133,134] and enhances the survival of ILCs [135]. In more detail, in mild asthmatic patients, an increase in the number of IL-5- and IL-13-expressing ILCs after allergen inhalation challenge was reported. These ILCs were found to express high levels of TSLPR, revealing a strong connection between ILCs and TSLP in the airways [136]. In humans, TSLPR is expressed on mast cells stimulated with TSLP, along with IL-1β and TNF-α, and produce Th2 cytokines and CXCL8 and CCL1 chemokines [101,137,138]. Additionally, mast cells can secret heightened levels of TSLP after IgE cross-liking or IL-4 priming [139]. A recent study in humans demonstrated that TSLP can act on human peripheral blood CD14^+^ monocytes/macrophages and activate them via inducing the expression of CD80, a process that could be linked to the differentiation of myeloid DCs [140]. Notably, a mouse study showed that TSLP can induce the alternative activation of macrophages (M2 macrophages) during allergic inflammation [141]. Finally, in humans, activated platelets express RANK ligand (RANKL) to interact with TSLP-stimulated myeloid DCs and contribute to their maturation via RANKL-RANK pathway, promoting the differentiation of naïve T cells to Th2 cells [142,143].

### 2.3. Role of TSLP in Allergic Airway Inflammation

Numerous studies using murine models have provided major insights regarding the role of TSLP in the development of allergic asthma [94,119]. Studies using ovalbumin (OVA)-induced AAI showed increased TSLP mRNA expression in the airway, while administration anti-TSLP reduced airway inflammation, mucus production, inflammatory cell inflammation and IL-4, IL-5, IL-6 cytokine release in the BAL [144]. In the same studies, injection of microRNA-19b also reduced airway inflammation and remodeling by STAT3 signaling inhibition through TSLP downregulation. In studies using HDM-induced AAI, intranasal administration of anti-TSLP mAb attenuated AHR, airway inflammation and the levels of IL-4 and IL-5 cytokine release in the BAL [145]. Mechanistically, anti-TSLP prevented the loss and redistribution of E-cadherin and b-catenin in the HDM-induced asthmatic mice through the blockade of AKT signaling pathways. A recent study demonstrated that exposure to particulate matter (PM2.5) augments AAI in a TSLP-related manner, illustrated by heightened TSLP levels in the lung following co-exposure to OVA and increasing doses of PM2.5 [146]. Mice lacking TSLPR (*Crlf2^−/−^* mice) are unable to generate robust Th2 cell effector responses and fail to develop airway inflammation to inhaled allergen, unless supplemented with wild-type allergen-primed CD4^+^ T cells [147,148,149]. In line with the aforementioned studies, adoptive transfer of allergen-primed TSLPR-deficient Th2 cells to recipient mice before antigenic challenge resulted in reduced airway eosinophilia and allergen-specific serum IgE levels compared to mice that received WT, allergen-primed Th2 cells, pointing towards a crucial role of this cytokine in Th2 memory-recall responses [150]. Notably, a very elegant study showed, by using multiple cell lineage-specific TSLPR-deficient mice, that TSLP displays distinct effects in models of airway inflammation depending on whether it is acting on cells of the innate or adaptive immunity branch [151]. Recent studies showed that co-exposure to HDM and diesel exhaust particles (DEP) induced an increase in BAL eosinophil, neutrophils, macrophages and CD4^+^ T-cell levels, compared to exposure to HDM alone [152]. Moreover, TSLPR deficiency decreased the number of eosinophils in the BAL and lung tissue upon HDM + DEP exposure, while it did not affect AHR. HDM + DEP co-exposure resulted in increased IL-13 levels in the lungs of WT mice in contrast to TSLP receptor-deficient mice [152], suggesting that TSLP partially mediates type 2 inflammation in this model of pollution-induced severe allergic airway disease.

### 2.4. Role of TSLP in Human Asthma

Increased expression of TSLP was observed in the airway lamina propria of SA patients. Genome-wide association studies showed that the TSLP single nucleotide polymorphism (SNP) rs1837253 positively correlated with childhood-onset asthma risk [153], while the same TSLP SNP was also identified as a susceptibility locus for adult asthma [154]. Increased expression of TSLP was observed in the airway lamina propria of SA patients [155,156,157] and, more importantly, was predictive of future disease exacerbations [158]. In addition to that, TSLP levels in BAL from asthmatics positively correlated with the number of neutrophils [157]. Moreover, several studies have shown enhanced TSLP gene expression in the asthmatic airway mucosa and increased TSLP levels in the BAL of patients with moderate-to-severe asthma [155]. Notably, bronchial allergen challenge led to significantly heightened expression of TSLP in the bronchial epithelium and submucosa of mild asthmatics and correlated with the extent of late-phase airway obstruction [114]. Importantly, increased levels of IL-4, a cytokine that enhances the permeability of airway epithelial cells, resulted in amplified TSLP levels and subsequent propagation of Th2 inflammatory responses [114]. In other studies, increased expression of TSLP receptor on alveolar macrophages from asthmatics correlated with longer disease duration and impaired lung function [159]. Stimulation of the 16HBE human bronchial epithelial cell line with HDM caused a significant increase in protein expression levels of TSLP, resulting in delocalization of E-cadherin [145]. In the THP-1 human cell line, treatment with TSLP induced ROS production, promoted mitochondrial complex activity, and increased mitophagy [160]. Other studies have shown that exposure of HBECs to DEP induced TSLP secretion [161]. Furthermore, in vitro, monocyte-derived dendritic cells co-cultured with DEP-treated HBECs exhibited a pro-Th2 phenotype characterized by increased surface expression of OX40 ligand and enhanced capacity to induce IL-5 production by CD4^+^ T-cells [161]. Additionally, other alarmins, such as High mobility box 1 protein (HMGB1), were found to be elevated in the sputum and serum of children with SA, compared to those with MMA and healthy individuals, and upon CS treatment, a significant reduction in this alarmin was observed. Notably, HMGB1-treated HBECs secrete increased amounts of TSLP, highlighting the interconnection of these two alarmins [162,163,164].

The fundamental role of TSLP in asthma pathogenesis was underscored by the fact that in 2021, US FDA approved tezepelumab (Tezspire), a human monoclonal antibody (IgG2λ) that inhibits the interaction of TSLP with its heterodimeric receptor, for SA treatment [149]. The therapeutic potential of tezepelumab was documented in several clinical trials. The first phase 1b randomized, double-blind, controlled trial that assessed the efficacy of tezepelumab in patients with asthma was reported in 2014 (Table 1) [165]. Tezepelumab significantly inhibited the decline of FEV1 and methacholine-induced airway hyperresponsiveness. In a phase II trial (the CASCADE trial; NCT03688074), administration of tezepelumab in patients with moderate-to-severe asthma led to a significant reduction in airway submucosal eosinophils in bronchial biopsies, the number of peripheral blood eosinophils, AHR and disease exacerbation versus placebo treatment [166]. Furthermore, in a phase IIb trial (the PATHWAY trial; NCT02054130), tezepelumab decreased disease exacerbations and improved lung function, asthma control and health-related quality of life of patients with severe, uncontrolled asthma compared with placebo [167,168]. Likewise, in a phase III trial (the NAVIGATOR trial; NCT03347279), asthma exacerbations rate were substantially lowered with tezepelumab compared with placebo in patients with severe, uncontrolled asthma, irrespective of low eosinophil numbers in the periphery at baseline. In addition, lung function was ameliorated, and less hospitalization and emergency room visits for patients treated with tezepelumab were observed [169]. Notably, in DESTINATION (NCT03706079), a long-term, randomized, placebo-controlled extension study, administration of tezepelumab for a two-year period was well-tolerated and achieved lasting and clinically profound declines in asthma exacerbations in individuals with severe, uncontrolled asthma [170].

In the CASCADE trial [166], the significant reduction observed in the numbers of airway submucosal eosinophils, IgE serum levels, as well as of other type 2-associated biomarkers, such as FeNO, IL-5 and IL-13 concentrations, suggest a tezepelumab effect mainly on type 2-mediated airway inflammation. Nevertheless, data from other phase 2 and 3 clinical trials reveal that administration of tezepelumab led to significant reductions in asthma exacerbations also in patients without evidence of type 2 inflammation [167,169], suggesting that tezepelumab could act through mechanisms beyond type 2 inflammation that remain to be determined. Indeed, the unanticipated efficacy of tezepelumab observed in patients with non-type 2, severe, uncontrolled asthma remains to be determined, as animal experiments have solely used so far Th2-dominated murine models of experimental asthma that do not recapitulate the complex type 1 and 17 inflammatory responses that prevail in the airways of severe asthmatics [27,171]. Furthermore, the improvements in asthma clinical outcomes observed in patients that received tezepelumab were primarily ascribed to substantially reduced eosinophilic airway inflammation, as submucosal eosinophils were the only inflammatory cells affected in terms of decreased percentages (the CASCADE trial; NCT03688074). Notably, other inflammatory cells such as group 2 ILCs whose effector properties and survival are largely associated with TSLP [135], were not measured in the above study. In addition, as tezepelumab treatment had no effect on airway remodeling and no improvement in spirometry or frequency of exacerbations were observed in this patient cohort, further investigation is required in order to dissect which cell types directly respond to TSLP in vivo and as a result influence downstream inflammatory responses that dominate in SA.

## 3. Conclusions

Several biologic therapies that target specific downstream effector molecules involved in asthma pathophysiology have been approved for patients with moderate-to-severe allergic and/or eosinophilic asthma. Nonetheless, none of them has shown superior efficacy in SA patients. Hence, targeting factors that hold broader effects on airway inflammation than existing biologics could constitute an attractive therapeutic approach for asthmatics who are unresponsive to currently available therapeutic regimes. One such appealing factor is TSLP, an important upstream initiator and mediator of the inflammatory cascade, whose therapeutic targeting was recently approved by FDA for SA. Importantly, initial results from the aforementioned clinical studies reveal that SA patients significantly benefit from tezepelumab treatment. Nevertheless, the cellular and molecular mechanisms accounting for its therapeutic capacity in non-type 2, severe, uncontrolled asthma remain poorly defined. Delineation of the mode of function of TSLP in SA represents an important piece of knowledge that will significantly advance SA management.

## Figures and Tables

**Table 1:** Clinical trials of Tezepelumab administration in asthma patients.

Study Title	Study Identifier	Study Phase	Outcome	Refs.
**Double-blind, Multiple Dose Study of Tezepelumab (AMG 157) in Adults With Mild Atopic Asthma**	NCT01405963	Ib	Attenuation of FEV1 decline Inhibition of methacholine-induced airway hyperresponsivenes	[165]
**Study to Evaluate Tezepelumab on Airway Inflammation in Adults With Uncontrolled Asthma (CASCADE)**	NCT03688074	II	Reduction of: airway submucosal eosinophils in bronchial biopsies peripheral blood eosinophils AHR disease exacerbation	[166]
**Study to Evaluate the Efficacy and Safety of MEDI9929 (AMG 157) in Adult Subjects With Inadequately Controlled, Severe Asthma (PATHWAY)**	NCT02054130	IIb	Reduction of asthma exacerbations Improvement of lung function Improvement of asthma control and health-related quality of life of patients	[167,168]
**Study to Evaluate Tezepelumab in Adults & Adolescents With Severe Uncontrolled Asthma (NAVIGATOR)**	NCT03347279	III	Reduction of asthma exacerbations Amelioration of lung function Reduction of hospitalization and emergency room visits	[169]
**Extension Study to Evaluate the Safety and Tolerability of Tezepelumab in Adults and Adolescents With Severe, Uncontrolled Asthma (DESTINATION)**	NCT03706079	III	Reduction of the annualised asthma exacerbation rate	[170]

## Data Availability

Not applicable.

## Abbreviations

AAI	allergic airway inflammation
HMGB1	High mobility box 1 protein
AHR	airway hyperresponsiveness
AEs	adverse effects
ASM	airway smooth muscle
BAL	bronchoalveolar lavage
CS	corticosteroid
CXCL8/CXC	chemokine IL-8
DCs	dendritic cells
DEP	diesel exhaust particles
FeNO	Fractional exhaled Nitric Oxide
HBECs	human bronchial epithelial cells
HDM	house dust mites
HMGB1	High mobility box 1
ILC2s	innate lymphoid cells type 2
MMA	mild-moderate asthmatics
NLRs	NOD-like receptors
OVA	ovalbumin
OX40L	OX40 ligand
PBMCs	peripheral blood mononuclear cells
PGA	paucigranulocytic asthma
PGD2	prostaglandin D2
PGRN	progranulin
PM2.5	particulate matter
RANKL	RANK ligand
RANTES	Regulated on Activation, Normal T cell Expressed and Secreted SA
RSV	respiratory syncytial virus
SNP	single nucleotide polymorphism
TARC	Thymus- and Activation-Regulated Chemokine Th2 T helper 2
Tfh	T follicular helper cells
TLRs	Toll-like receptors
TNF-α	tumor necrosis factor
Tregs	regulatory T cells
TSLP	thymic stromal lymphopoietin
